# Biphasic positive airway pressure spontaneous breathing attenuates lung injury in an animal model of severe acute respiratory distress syndrome

**DOI:** 10.1186/s12871-022-01763-w

**Published:** 2022-07-16

**Authors:** Leilei Zhou, Rui Yang, Chunju Xue, Zongyu Chen, Wenqing Jiang, Shuang He, Xianming Zhang

**Affiliations:** 1 School of Clinical Medicine, Guizhou Medical University, Guiyang, 550004 China; 2 Department of Internal Medicine, Guiyang First People’s Hospital, Guiyang, China; 3 Department of Respiratory and Critical Care Medicine, The Affiliated Hospital of Guizhou Medical University, Guiyang, 550004 China

**Keywords:** Acute respiratory distress syndrome, Inspiratory muscles, Spontaneous breathing, Biphasic positive airway pressure, Neuromuscular blocker

## Abstract

**Objective:**

To compare the effects of unassisted spontaneous breathing (SB) and complete muscle paralysis (PC) on early severe acute respiratory distress syndrome (ARDS) in an animal model, and to explore the possibility of biphasic positive airway pressure (BIPAP) as lung protective ventilation support for patients in the early stage of severe ARDS.

**Methods:**

Twelve healthy beagle dogs between the ages of 10 and 15 months were randomly divided into two groups: the SB group (BIPAP_SB_) and the PC group (BIPAP_PC_). Arterial blood samples were drawn before modelling. Arterial blood gas analysis and mechanical tests were conducted. The animal model of severe ARDS was established using a deep intravenous injection of oleic acid, and BIPAP ventilation was performed for 8 hours. Lung tissue and blood were taken to detect lung function, inflammatory reactions and degree of pathological damage.

**Results:**

At the beginning of the experiment, there was no significant difference in the arterial blood gas analysis between the two groups (*p* > 0.05). After successful modelling, the oxygenation index and the end-expiratory lung volume in the SB group were significantly higher than those in the PC group 8 hours after MV. Pathologically, the wet-dry ratio and pathological score of the PC group were higher than those of the SB group; the lung injury in the gravity-dependent area in the SB group was less than that in the PC group (*p*< 0.05).

**Conclusions:**

In the early stage of severe ARDS induced by oleic acid, compared with PC, retention of the BIPAP mode of SB can reduce the risk of lung injury and improve respiratory function.

## Introduction

The novel coronavirus disease 2019 (COVID-19) has spread throughout the world. COVID-19 mainly involves the respiratory system, and there is less damage to other organs. Studies have shown that critically ill patients can be complicated with acute respiratory distress syndrome, with an incidence rate of 15.6–31% [1, 2]. Mechanical ventilation is the main supportive treatment of ARDS, and improved ventilation methods, such as biphasic positive airway pressure (BIPAP), significantly improved the survival rate of ARDS patients. Based on the open lung strategy, BIPAP is a time-switched, pressure-controlled ventilation mode that can superimpose fully spontaneous breathing (SB) at two different levels of continuous positive airway pressure (CPAP) without confrontation. In this ventilation mode, SB can occur at any stage of the ventilation cycle. BIPAP ventilation is a ventilation mode that can prevent and reduce the occurrence of ventilator-induced lung injury (VILI) [3, 4].

In ARDS, retention of SB is associated with better ventilation and less atelectasis in the gravity-dependent areas of the lungs and less overexpansion of the gravity-dependent areas of the lungs. In addition, the retention of SB can prevent periodic alveolar collapse, which may reduce atelectasis and may be associated with shorter intensive care unit stays [5–7]. Jin et al [8] found that in mild to moderate ARDS animal experiments, compared with controlled mechanical ventilation, SB reduced the pathological injury of lung tissue. However, some scholars believe that SB is unable to control tidal volume, which is prone to barotrauma; SB is not synchronized with ventilators to produce man-machine confrontation, which increases discomfort in the patient; SB can lead to lung hyperinflation, produce endogenous positive end-expiratory pressure (PEEP) and increase the expression of inflammatory mediators, resulting in VILI [9–11]. In addition, Yoshida et al. [12] found that animal experimental studies have shown that SB may exacerbate a lung injury in severe ARDS patients, while neuromuscular blockers can alleviate a lung injury. NMBAs (neuromuscular blocking agents) have been used in ARDS treatment for decades, and are used to help increase alveolar recruitment and improve patient synchronization with the ventilator when administered in critically ill patients with ARDS within 48 hours after lung injury. In addition, NMBAs promote oxygenation and reduce mortality by preventing respiratory muscle contraction [13–15]. In 2010, ACURASYS, a large multicentre trial, indicated that early use of neuromuscular blockers in patients with severe ARDS (oxygenation index, OI, PaO_2_/FiO_2_ < 150) could shorten mechanical ventilation time, reduce organ failure and mortality [16]. However, in moderate to severe ARDS patients treated with a high PEEP strategy, recent studies showed that there was no significant difference in 90-day mortality between patients who received an early and continuous cisatracurium infusion and those who received routine care and mild sedation [17]. Whether NMBAs are routinely used for patients in the early stage of severe ARDS is controversial [18]. Moreover, it has been proven that muscle relaxation is superior to SB in the early stage of severe ARDS. The ventilation mode used is volume-assisted-control ventilation (A/C-V) and pressure-assisted-control ventilation (A/C-P), which is an auxiliary autonomous breathing mode. This mode results in a higher probability of respiratory superposition, man-machine asynchrony and a greater possibility of mechanical VILI [19]. Therefore, it is not appropriate to completely extrapolate the experimental results of the assisted SB mode to the unassisted SB mode. It is important to study the protective effect of unassisted autonomous breathing modes, such as BIPAP, on patients in the early stage of severe ARDS and its comparison with fully controlled ventilation in severe acute respiratory distress syndrome. In this study, we hypothesized that in the critical ARDS model induced by oleic acid inhalation, the use of the BIPAP unassisted SB mode could further improve oxygenation and alleviate VILI when compared with NMBAs.

## Methods

### Animal Preparation

The use of experimental animals complies with the requirements of Chinese regulations on the Administration of Experimental Animals and has been approved by the Animal Experimental Ethical Inspection Form of Guizhou Medical University. The study included twelve healthy beagle dogs, male and female, that weighed between 10 and 14 kg and were between the ages of 9 and 15 months. A combination of pentobarbital sodium (30 mg/kg) and diazepam (5 mg) was injected intramuscularly to induce anaesthesia, and then propafenone (2–4 mg/kg/h) was injected intravenously to maintain anaesthesia. In addition, cisatracurium 0.16 mg/kg was injected intravenously to completely relax spontaneous breathing in the beagle dogs. The beagle dog was fixed on the animal operating table in the supine position and intubated through the mouth to ensure that the airway was unobstructed. The dog was ventilated with an Evita4 ventilator (Dräger, Germany), and the electric blanket was opened. The body temperature was 37 ± 1 °C. Integrated myoelectric tubes of the esophageal sac, gastric sac and esophageal diaphragm were implanted, and the airway pressure, esophageal pressure and intragastric pressure were recorded. Respiration, heart rate and blood oxygen saturation were monitored by the ECG monitor. After 15 minutes of stability, the physiological data were collected under normal conditions. After 15 minutes of stability, the beagle dogs were ventilated in IPPV mode, with a tidal volume (VT) of 10 ml/kg, a positive end-expiratory pressure (PEEP) of 5 cmH_2_O; the inspiratory/expiratory ratio (IE) was 1:1.5, the flow rate was 20 L/min and the respiratory rate (RR) was 20 breaths/min, as adjusted according to the arterial blood gas analysis, and PaCO_2_ was maintained between 35 and 45 mmHg.

### Experimental protocol

After full mixing of 40 ml normal saline and total dose of 0.3 ml/kg oleic acid, it was slowly infused into the right atrium through the inferior vena cava catheter through the infusion pump. The infusion rate was 40 ml / kg. The artirial blood gas analysis was performed 90 minutes after infusion. When OI < 100 mmHg and stabilized for 30 minutes. It shows that the serious ARDS model is manufactured successfully [20, 21]. After inducing lung injury, the lung was reopened, using BIPAP mode. After that, 12 animals were randomly divided into PC group (BIPAP+PC group, n = 6) and SB group (BIPAP+SB group, n = 6).cisatracurium 0.16 mg/kg was injected intravenously to completely relax the spontaneous breathing of beagle dogs, and the dogs were ventilated with BIPAP for 8 hours. In the SB group, the inspiratory / expiratory ratio of FiO_2_:100%, phigh: was titrated at the time of VT 6 ml/kg, plow:10cmH_2_O, PaCO2:30 breaths/min in the beginning. After completion of the setting, cisatracurium, a neuromuscular blocker were stopped, and the respiratory rate of controlled ventilation was reduced to 15 breaths / min. According to the artirial blood gas analysis, the respiratory rate of the ventilated part was adjusted at 1:1 according to the blood gas analysis to ensure that the respiratory rate of the ventilated part was between 35 and 60 mmHg. The PC group continued to use neuromuscular blockers, and RR was ventilated at the beginning of 30 times per minute at any time, and other ventilator settings were the same as those in the SB group.

### Measurements

Hemodynamics, oxygenation index, VD/VT, EELV and respiratory mechanics of the two groups of beagle dogs were measured every 2 hours. The transpulmonary pressure (PL) is calculated from the difference between the airway pressure (Paw) and the esophageal pressure (Peso). Under the BIPAP ventilation mode, the average paw can be calculated as: (Phigh × Ttigh+Plow × Tlow) / (Ttigh+Tlow), where Ttigh is the time length of Phigh and Tlow is the time length of Plow. EELV of dogs in each group was measured by end-expiratory breath-holding method at the beginning of the experiment, at the end of the model and at the end of the experiment. VD/VT was calculated by end-expiratory carbon dioxide partial pressure (PetCO_2_) method according to Enghoff equation: VD/VT = (PaCO_2_-petCO_2_) / PaCO_2_ [22].

### Protein expression levels of inflammatory mediators

The pro-inflammatory mediators expressed before, during and at the end of the experiment. 3 ml of mixed venous non-anticoagulant blood was extracted from the deep venous catheter, coagulated at room temperature for 2 hours, and centrifuged at the rate of 2000 rpm for 15 min. The serum EP tube was taken and stored in the refrigerator at-80 °C for detection. Interleukin 6 (IL-6) and interleukin 8 (IL-8) were selected and the protein levels of which were determined by enzyme-linked immunosorbent assay kit (Guangzhou Jikun Biological Co.Ltd.China.). All enzyme-linked immunosorbent assay procedures were carried out according to the manufacturer’s plan.

### Lung histopathology

At the end of the experiment, the experimental animals were anesthetized with 3% pentobarbital and propofol, and 10% potassium chloride injection 10-15 ml was injected intravenously to carry out euthanasia. After euthanasia, the lungs were washed with normal saline, and the blood and water on the surface of the lungs were quickly dried with filter paper. Separate the right lung tissue. After weighing the wet weight, it was placed in wide-mouth glassware and baked at 60 °C for 48 hours to constant weight, and then weighed again. The average ratio of wet to dry of 5 lung tissues represents the ratio of wet to dry of the whole lung tissue [12]. Tissue sections were stained with HE. Based on the scoring system reported in the previous literature, eleven representative lesions were selected to score [23].

### Statistical analysis

SPSS25.0 statistical software was used for statistical analysis. The data were expressed as mean ± standard deviation. The wet-dry ratio of the two groups was compared by two independent samples t-test. One-way ANOVA was used for comparisons between experimental groups. For multiple testing, Tukey analysis was performed. The respiratory mechanics indexes were compared by using two-way repeated measures ANOVA with group and time. GraphPad Prism8. 3 was used to drawing and perform statistical analyses. A *P* value of < 0. 05 was considered significant.

## Results

### Haemodynamics, ventilatory, gas exchange, and respiratory mechanics

There was no significant differences in HR, mean arterial pressure (MPA), OI or respiratory mechanics parameters between the two groups before the respiratory mechanics models were established. After modelling, the gas exchange deteriorated, and the oxygenation index decreased below 100 mmHg. In addition, the OI values of each experimental group were significantly different from those before modelling (*p*< 0.05) (Table 1).

**Table 1:**
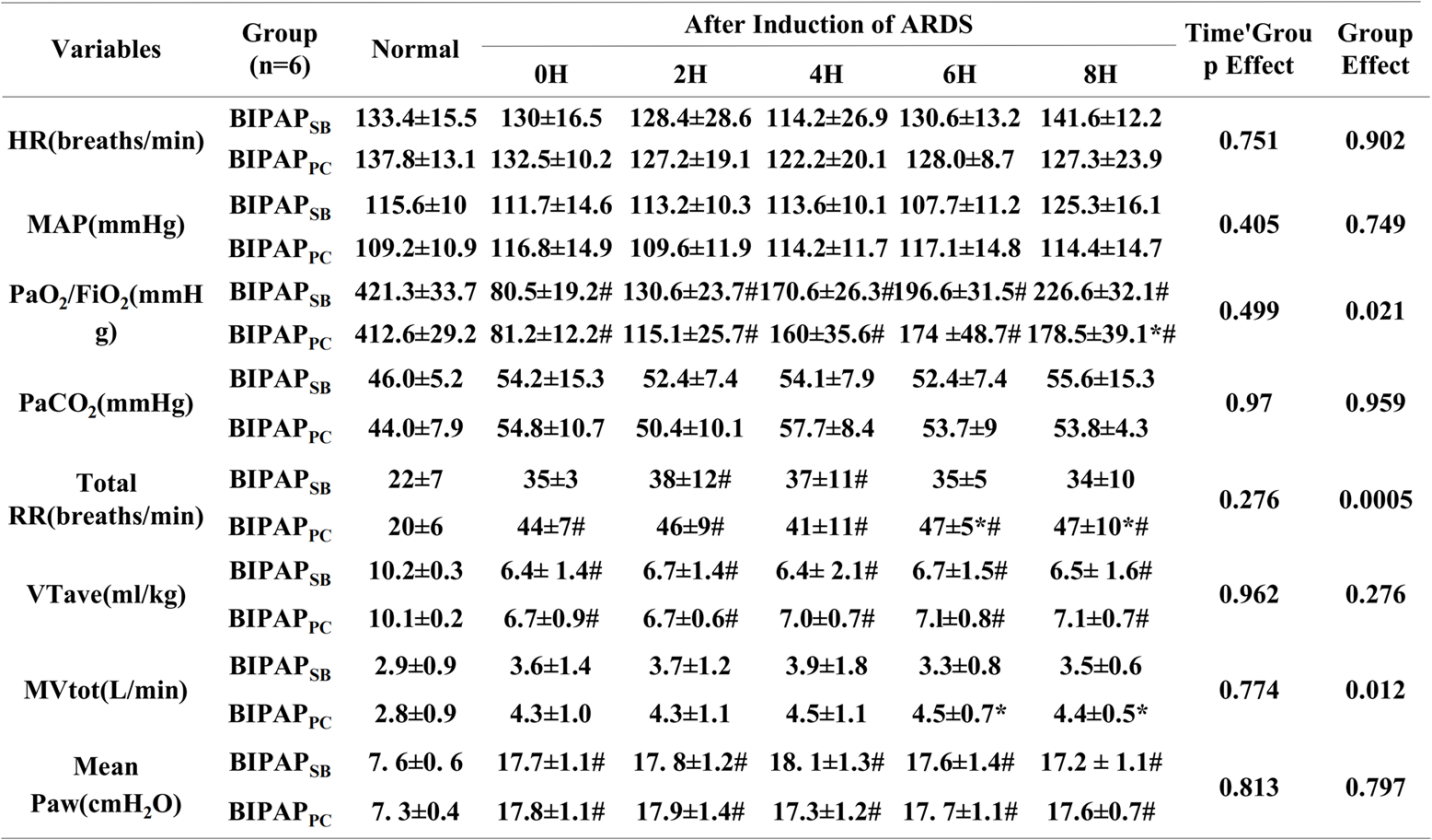
Hemodynamics and Respiratory Measurements

Values are means ± SD. *BIPAP*_*SB*_ Biphasic positive airway pressure with SB, *BIPAP*_*PC*_ Biphasic positive airway pressure with muscles paralysis, *SB* Spontaneous breathing, *MV* Minute ventilation; Normal: beagle dogs not injected with oleic acid0H:0H is the state of beagle dogs injected with oleic acid without BIPAP ventilation.; *HR* Heart rate, *PaCO*_*2*_ Partial pressure of carbon dioxide, *MAP* Mean arterial pressure, *PaO*_*2*_*/FiO*_*2*_ ratio of partial pressure of arterial oxygen to faction of inspired oxygen concentration, *RR* Respiratory rate, *VTave* Average tidal volume, *Pplat* Plateau pressure, *PTP* Pressure time product, *mean Paw* mean airway pressure, *peak PL* peak transpulmonary pressure, *mean PL* mean transpulmonary pressure, *Peso = esophageal pressure* Δ Pes = change of esophageal pressure, *Pgas* intragastric pressure.*p < 0.05, compared with BIPAP_SB_ group, #*p* < 0.05, compared with Normal group

**Fig. 1:**
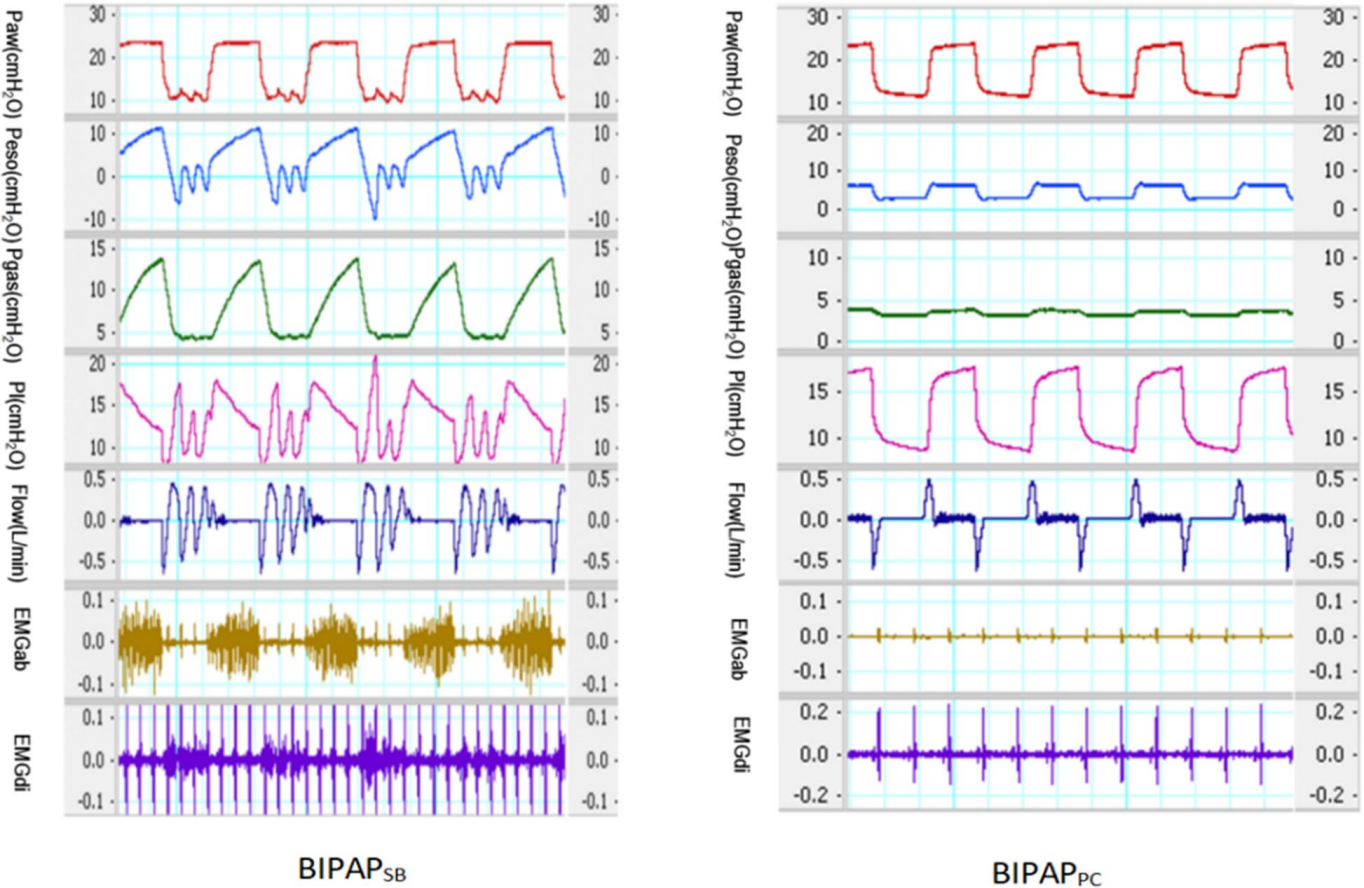
Representative respiratory tracings of airway pressure (Paw), esophageal pressure (Pes), intragastric pressure (Pgas), transpumonary pressure (PL), Airflow, abdominal muscles surface electromyography (EMGab) and diaphragmatic esophageal surface electromyography (EMGdi) in BIPAP_SB_, BIPAP_PC_ group in representative animals. BIPAP_SB_ = biphasic positive airway pressure with spontaneous breathing, SB efforts were regained; BIPAP_PC_ = biphasic positive airway pressure with muscles paralysis, Animals’ SB efforts were fully depressed. Therefore, BIPAP was equal to pressure-controlled ventilation

The respiratory mechanics curves of the BIPAP_SB_ group and BIPAP_PC_ group in BIPAP mode are shown in Fig. 1. As seen in the picture, the main difference between the groups was whether they had respiratory muscle activity. Diaphragm EMG and abdominal EMG were observed at the same time in the BIPAP_SB_ group but disappeared completely in the BIPAP_PC_ group. In addition, the gastric pressure and esophageal pressure in the PC group were significantly lower than those in the SB group. The pressure-time curve showed that spontaneous breathing appeared in BIPAP_SB_ group, and spontaneous breathing mainly occurred in low pressure. In BIPAP_PC_ group, there was no spontaneous breathing, which was a typical curve of pressure controlled ventilation. The curves of gastric pressure and esophageal pressure also showed that there was no notch of negative change in spontaneous breathing in BIPAP_PC_ group and negative notch in spontaneous breathing in BIPAP_SB_ group.

**Fig. 2:**
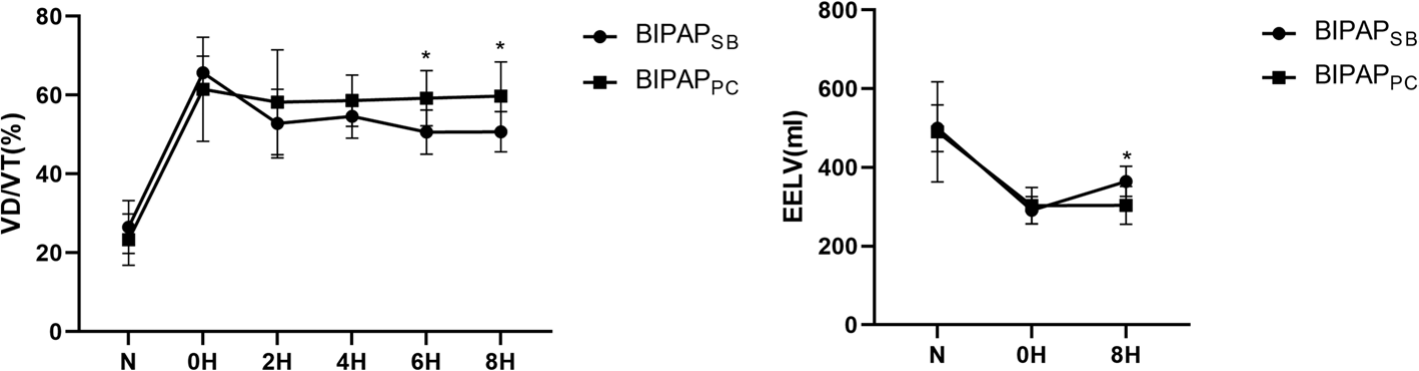
Time course of the end expiratory lung volume (EELV) and VD/VT in experimental groups (*n* = 6 per group). N=Pre-modeled animal model; BIPAP_SB_ = biphasic positive airway pressure with spontaneous breathing; BIPAP_PC_ = biphasic positive airway pressure with muscles paralysis; *p< 0.05, vs. BIPAP_SB_ group

As shown in Fig. 2, the EELV in the BIPAP_SB_ group was higher than that in the BIPAP_PC_ group. In addition, VD/VT in the BIPAP_SB_ group tended to be lower than that in the BIPAP_PC_ group after 2 hours of ventilation, and there was a significant difference after 6 hours (*p* < 0.05) (Fig. 2). The oxygenation index of the BIPAP_SB_ group was significantly higher than that of the BIPAP_PC_ group after 6 hours of ventilation (Table 1).

### Protein expression levels of inflammatory mediators

**Fig. 3:**
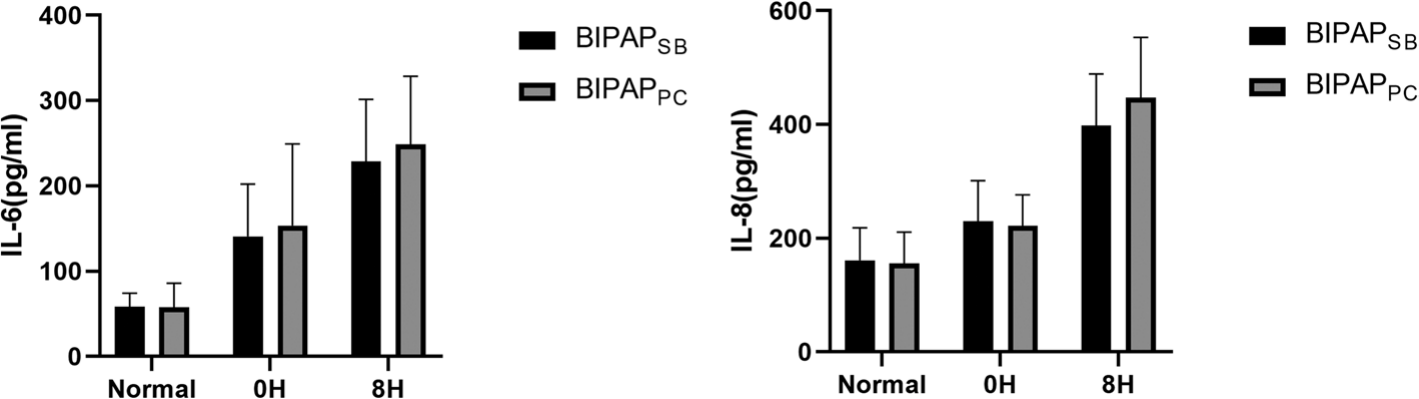
The Levels of interleukin (IL)-6 and IL-8 in plasma after 8 h mechanical ventilation. BIPAP_SB_ = biphasic positive airway pressure with spontaneous breathing; BIPAP_PC_ = biphasic positive airway pressure with muscles paralysis

As shown in Fig. 3 and Table 2, there were no significant differences in plasma IL-6 and IL-8 levels between the two groups before and after lung injury. After 8 hours of MV treatment, the plasma IL-6 and IL-8 levels in the BIPAP_SB_ group and BIPAP_PC_ group were significantly higher than those before modelling, and the plasma IL-6 and IL-8 levels in the BIPAP_SB_ group were lower than those in the BIPAP_PC_ group; however, there was no significant difference (*p* > 0.05).

**Table 2:**
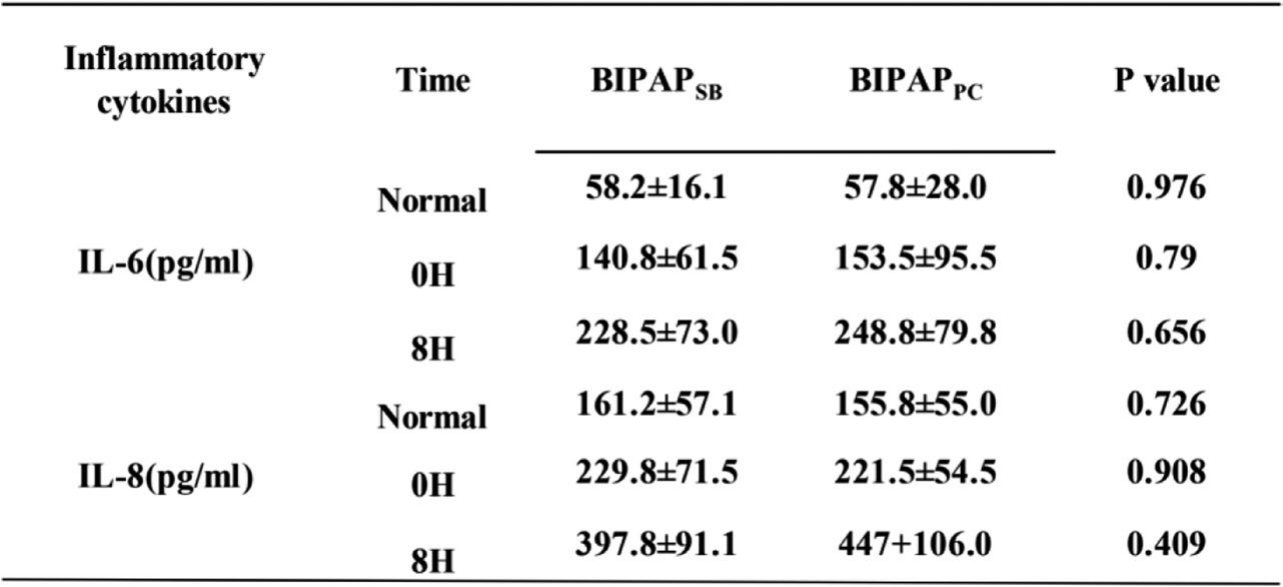
The Levels of interleukin (IL)-6 and IL-8 in plasma after 8 h mechanical ventilation

*BIPAP*_*SB*_ Biphasic positive airway pressure with SB, *BIPAP*_*PC*_ Biphasic positive airway pressure with muscles paralysis. Normal: beagle dogs not injected with oleic acid. 0H:0H is the state of beagle dogs injected with oleic acid without BIPAP ventilation

### The ratio of wet to dry in the lung

**Fig. 4:**
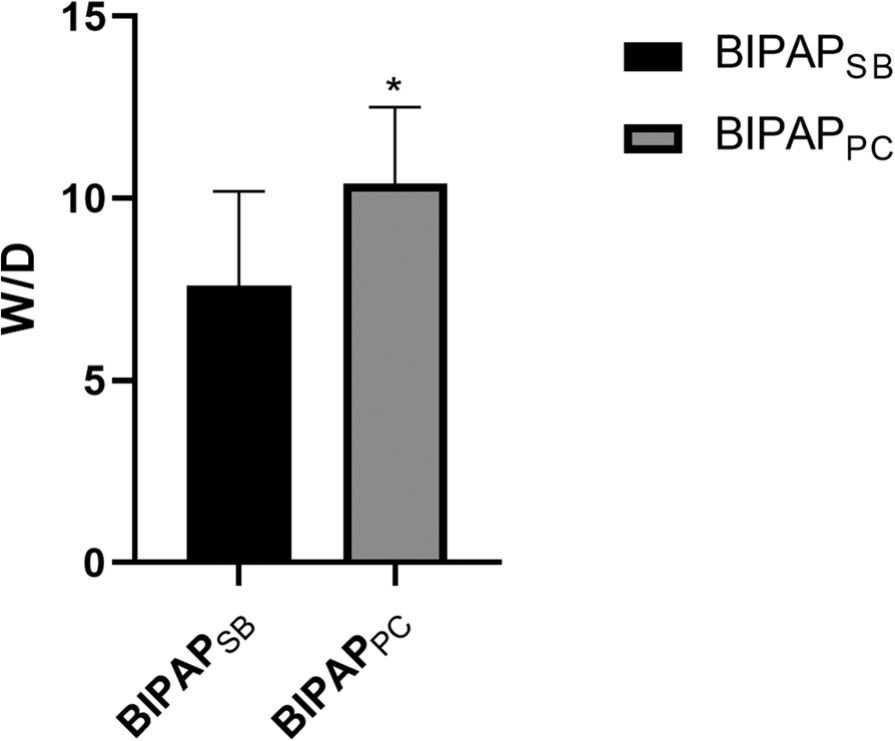
The Levels of Wet to dry weight ratio (W/D) after 8 h mechanical ventilation. BIPAP_SB_ = biphasic positive airway pressure with spontaneous breathing; BIPAP_PC_ = biphasic positive airway pressure with muscles paralysis;*p< 0.05 vs. BIPAP_SB_

As shown in Fig. 4, the ratio of wet to dry in the BIPAP_PC_ group was higher than that in the BIPAP_SB_ group (*p*< 0.05).

### Lung histopathological injury

**Fig. 5:**
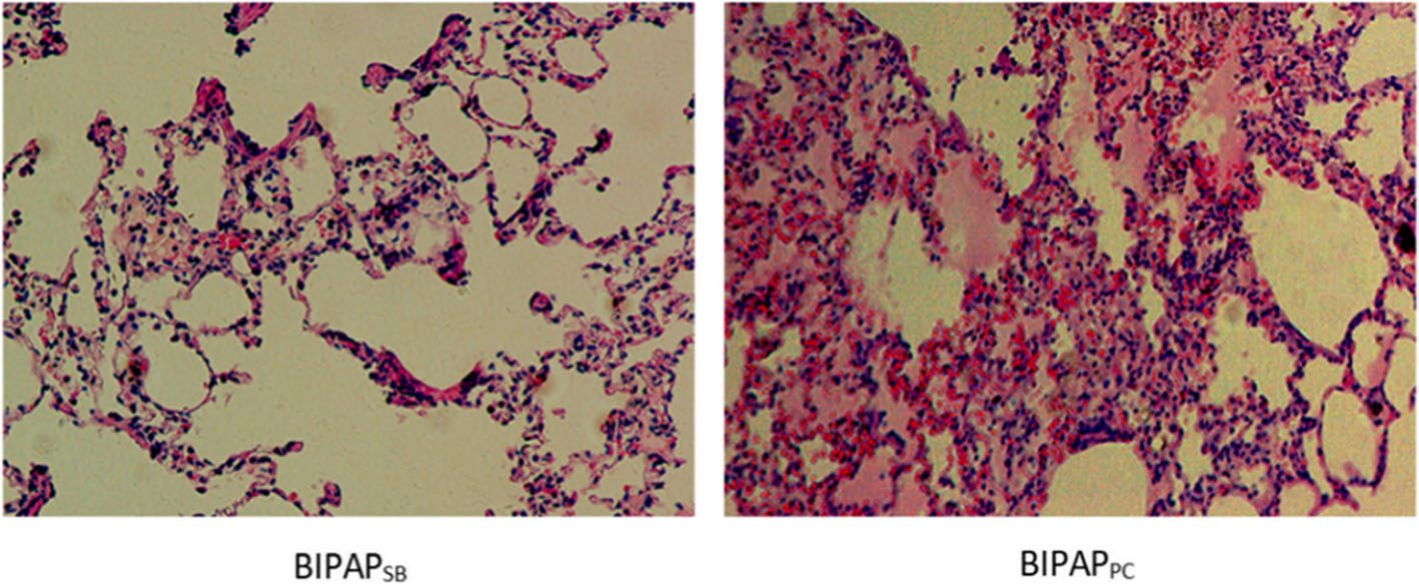
Representative appearances and photomicrographs of hematoxylineosin stained lung sections (magnification× 400) from in BIPAP_SB_ (A), BIPAP_PC_ group(B) in representative animals. BIPAP_SB_ = biphasic positive airway pressure with SB; BIPAP_PC_ = biphasic positive airway pressure with muscles paralysis

Compared with the BIPAP_PC_ group, the BIPAP_SB_ group had less lung injury, less alveolar bleeding, less hyperaemia and less neutrophil infiltration. The BIPAP_PC_ group showed more severe alveolar bleeding, edema, more inflammatory cell infiltration and thicker alveolar walls (Fig. 5). The total lung histopathology score in the BIPAP_SB_ group was significantly lower than that in the BIPAP_PC_ group (*p* < 0.001) (Table 3).

**Table 3:**
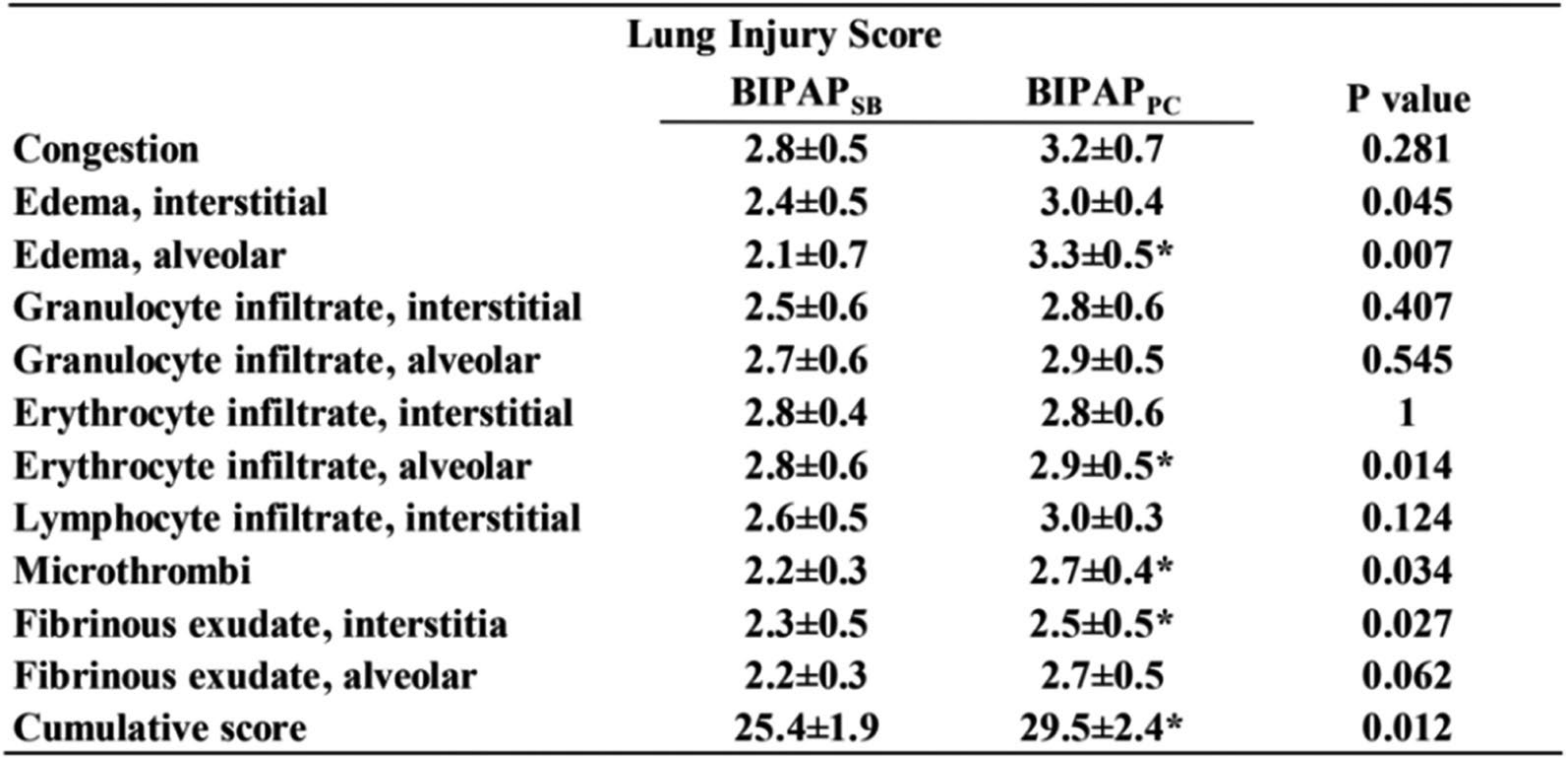
Histological sub-scores in experimental groups

Values are means±SD. *BIPAP*_*SB*_ Biphasic positive airway pressure with SB, *BIPAP*_*PC*_ Biphasic positive airway pressure with muscles paralysis, *SB* Spontaneous breathing; Grading as: 0, minimal changes; 1, mild; 2, moderate; 3, severe; 4, maximal changes

## Discussion

In an experimental model of the early stage of severe ARDS, our study showed that BIPAP ventilation with SB effectively improved gas exchange. Compared with muscle relaxation, BIPAP_SB_ effectively reduced the pathological injury of lung tissue. To better control the experimental conditions, we chose oleic acid to establish the ARDS model. The model has similarities regarding the basic clinical features of ARDS patients [21, 24]. There are many methods of mechanical ventilation for ARDS, and the reasons why we choose the BIPAP ventilation mode are as follows: First, the BIPAP ventilation mode is different from the A/C-V mode in that it is an unassisted SB ventilation mode, which can occur at any stage of the mechanical ventilation cycle and is only related to the respiratory drive of the experimental animals themselves, The main feature of BIPAP is that SB can be allowed during any phase of the mechanical cycle, so it is easy to maintain comparable levels of ventilator support between BIPAP with SB and without SB. Second, studies have shown that pressure is the main factor of VILI, and the BIPAP ventilation mode easily adjusts the ventilation parameters to make the average airway pressure of each experimental group consistent so that the experiment is comparable.

Our study showed that, compared with muscle relaxation, oxygenation improved after 2 hours of unassisted SB, and there was a significant difference in oxygenation index at 8 hours. The mechanisms may be as follows: First, SB improves oxygenation by increasing EELV. Under the same mean airway pressure, we found that EELV increased significantly in the SB group when compared with the PC group. Douglas et al [25] showed that the increase in EELV was positively correlated with the improvement in oxygenation. Similar to our study, SB increased EELV and significantly improved its oxygenation index. Second, SB improves oxygenation by reducing VD/VT. VD/VT is the ratio of dead space volume to tidal volume. The main pulmonary lesions in patients with ARDS are alveolar collapse, atelectasis, consolidation, pulmonary vasospasm and microvascular thrombosis in the gravity-dependent area of the lung, so VD/VT increases during ARDS. Kleinman et al. found that the gases inhaled during SB were mainly distributed in gravity-dependent areas. In the supine position, due to the action of gravity, the pathological changes of the lungs gradually increase from the ventral to the dorsal side, and the diaphragm will move to the head due to the compression of the abdominal organs, so muscle relaxation will further aggravate the pathological changes in the gravity-dependent area, while retaining SB in the patient, which can improve the ventilation of the gravity-dependent area of the lung and increase the reflux of the blood in this area, thus the VD/VT is decreased and oxygenation is improved [26].

Mechanical ventilation can lead to VILI through a series of mechanisms [27]. Some animal experiments have shown that severe SB in ARDS patients may aggravate a lung injury, and neuromuscular blockers can alleviate a lung injury [21, 25]. However, it is worth noting that both studies used A/C-V and A/C-P ventilation modes, which are more likely to cause human–machine asynchrony and respiratory superposition, which produces gas traps. In a study of oleic acid-induced severe ARDS in beagle dogs, it was shown that, when compared with muscle relaxation, BIPAP ventilation with SB could reduce the pathological injury of the lung tissue, reduce the wet/dry weight ratio of the lung tissue, and significantly reduce the total score of a lung injury. The mechanisms may be as follows: First, reduce the strain value [28, 29]. Studies have shown that a high strain value may be one of the main indicators of a lung injury. If the strain value is equal to the VT/EELV value, then the lung injury is more severe. In this experiment, compared with the PC group, the VT/EELV value of the SB group was relatively lower, so the lung injury was alleviated. Second, the gas in the lungs was redistributed. There was a significant difference in the gas distribution in the lungs between the SB and PC groups. After SB redilates the solid alveoli in the gravity-dependent area, the gas in the lung is redistributed from the nongravity-dependent area to the gravity-dependent area. After the tidal volume is redistributed to the gravity-dependent area, a reduction in the hyperinflation state of the nongravity-dependent area is likely, so the lung injury is reduced. Third, the shear injury was reduced. Compared with muscle relaxation, this study found that unassisted SB can increase intrapleural negative pressure, thus resulting in increased transpulmonary pressure. Transpulmonary pressure first affects the lung region near the diaphragm, and then alveolar re-expansion reduces recurring opening and closing of the alveoli in the gravity-dependent areas, thereby reducing the shear injury [30].

There are also some shortcomings in this study. First, there was no statistical significance in the expression of IL-6 and IL-8 in the plasma between the two groups, and there was no further study on whether there was statistical significance in the RNA level between the two groups. Second, abdominal muscle activity was obvious during strong SB, thus resulting in high intra-abdominal pressure. Studies have shown that excessive intra-abdominal pressure can reduce transpulmonary pressure and may aggravate a lung injury that is caused by mechanical ventilation. Last, the respiratory frequency and nerve distribution in beagle dogs are different from those in humans. Whether the results of this experiment can be extrapolated for patients with ARDS needs further study.

In conclusion, in this oleic acid aspiration–induced lung injury model, we found that, compared with controlled protective mechanical ventilation, preserved SB during BIPAP attenuated lung inflammatory responses, lung histopathological injury and improved gas exchange. The latest research shows that neuromuscular blockers cannot reduce the mortality of patients with moderate and severe ARDS within 90 days, which questions whether NMBAs should be routinely used or used as a rescue treatment in patients in the early stage of severe ARDS. Therefore, further study on the protective role of unassisted autonomous respiratory ventilation in patients in the early stage of severe ARDS is needed.

## Data Availability

The datasets used and analyzed during the current study are available from the corresponding author on reasonable request.
